# Protective Effect of Butanolic Fraction of *Delphinium brunonianum* on Fructose-Mediated Metabolic Alterations in Rats

**DOI:** 10.3390/metabo12060481

**Published:** 2022-05-26

**Authors:** Syed Nasir Abbas Bukhari, Hira Asif, Alamgeer, Mulazim Hussain Asim, Hafiz Muhammad Irfan, Hasan Ejaz, Mervat A. Elsherif, Kashaf Junaid

**Affiliations:** 1 Department of Pharmaceutical Chemistry, College of Pharmacy, Jouf University, Sakaka 72388, Al Jouf, Saudi Arabia; sbukhari@ju.edu.sa; 2 Lahore Pharmacy College, Lahore Medical and Dental College, Lahore 54850, Pakistan; 3 College of Pharmacy, University of Sargodha, Sargodha 40100, Pakistan; asimawan786pk@gmail.com (M.H.A.); irfan_pharmacist06@yahoo.com (H.M.I.); 4 College of Pharmacy, University of the Punjab, Lahore 05422, Pakistan; 5 Department of Clinical Laboratory Sciences, College of Applied Medical Sciences, Jouf University, Sakaka 72388, Al Jouf, Saudi Arabia; hetariq@ju.edu.sa (H.E.); kjunaid@ju.edu.sa (K.J.); 6 Chemistry Department, College of Science, Jouf University, Sakaka 72388, Al Jouf, Saudi Arabia; maelsherif@ju.edu.sa

**Keywords:** metabolic syndrome, oxidative stress, sympathetic over-activity, insulin resistance, LC-MS analysis

## Abstract

The present study was conducted with an intent to evaluate the protective effect of butanolic fraction of *Delphinium brunonianum* on fructose mediated metabolic abnormalities in rats. Rats in all groups except control group were fed on 10% fructose for 6 weeks; however, rats in the treated group also received butanolic fraction for the last 3 weeks, along with the fructose. Moreover, phytoconstituents present in butanolic fraction were analyzed using LC-MS. All doses of butanolic fraction profoundly reduce the fructose-induced blood pressure, sympathetic over-activity, and weight gain. Furthermore, butanolic fraction prominently reduces the glucose intolerance and hyperinsulinemia in fructose-fed rats. On treatment with butanolic fraction, oxidative enzymes and the functionality of the aorta was also restored. Phytochemical analysis revealed the presence of several active constituents including bergenin, scopolin, rutinoside, kaempferol, coumaric acid, apigenin, and gingerol. In conclusion, butanolic fraction of *Delphinium brunonianum* has the potential to prevent and recover the fructose-induced metabolic perturbations.

## 1. Introduction

Metabolic syndrome is characterized by a cluster of metabolic disorders including hypertension, hyperinsulinemia, dyslipidemia, and obesity. Metabolic syndrome is triggered by several genetic and environmental factors; however, the etiology of this syndrome is still unknown [1]. Currently, metabolic syndrome is a global health issue as each factor predisposes individuals to cardiovascular diseases and diabetes [2]. The use of herbal medicine is as old as the existence of man. Current appraises have propounded that, in many emerging countries, approximately 70–80% of the population reckons laboriously on medicinal plants in their vicinity to encounter their basic health care needs [3]. The literature showed that approximately four billion people around the world are using THM (traditional herbal medicine) for their healthcare needs [4]. In the contemporary world, the use of medicinal plants is of enormous public interest due to the increasing cost of allopathic drugs and associated ADRs. Furthermore, aboriginal plants serve as a source for the discovery of new drugs by providing templates for designing new drugs [5]. Several active constituents have been identified, isolated from medicinal plants, and are being used as a successful treatment option [6]. *Delphinium brunonianum* is distributed at the altitude range of 3500 to 6000 m in Pamir, Afghanistan, the Himalayan region, and south-east Tibet. Empirically, this species is being used for the treatment of several anomalies, including fever, headache, stomach ache, and for the purpose of blood purification [7,8]. Phytochemical investigation on this species has shown it to contain many valuable phytoconstituents [8,9]. Earlier, we reported the diuretic effect of *Delphinium brunonianum*, and crude extract has also been assessed for its effect on fructose-mediated hypertension and metabolic alterations [10,11]. Taking this into account, we further extended this study and evaluated the effects of butanolic fraction of *Delphinium brunonianum* on fructose-mediated perturbations. As we performed these two experiments at the same time in the same laboratory conditions, the results of the fraction-treated groups are compared with the same control group used for the evaluation of crude extract of *Delphinium brunonianum* [11].

## 2. Results

### 2.1. DB-B-Caused Decrease in Blood Pressure in Fructose-Fed Hypertensive Rats

**Figure 1:**
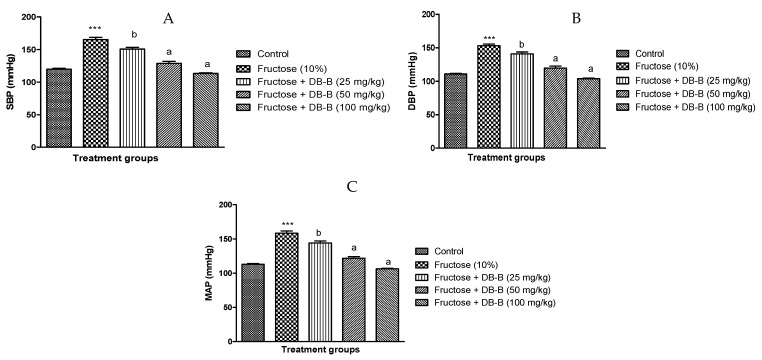
DB-B-mediated fall in blood pressure of fructose-fed hypertensive rats: (**A**) SBP (systolic blood pressure), (**B**) DBP (diastolic blood pressure), (**C**) MAP (mean arterial pressure). Results are expressed as mean ± SEM (n = 5). One-way analysis of variance was applied. *** = *p* < 0.001 when compared with the control group, whereas a = *p* < 0.001 and b = *p* < 0.01 in relation to the fructose (10%) group.

In the disease control group, elevation in blood pressure occurs in rats as compared with animals in control group. After treatment, the rats showed remarkable (*p* < 0.01–0.001) decreases in systolic, diastolic, and mean arterial pressure compared with the fructose-treated group, as shown in Figure 1.

### 2.2. DB-B-Induced Decrease in Sympathetic Over-Activity in Fructose-Fed Hypertensive Rats

**Figure 2:**
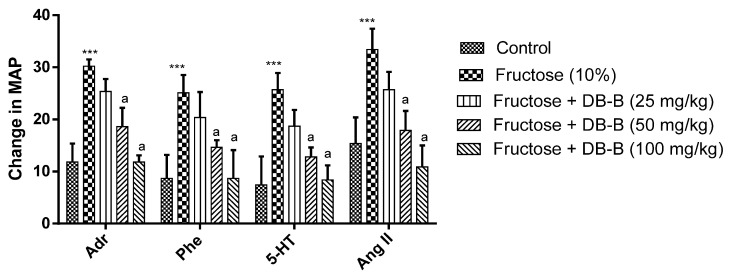
DB-B-evoked decrease in sympathetic activity in fructose hypertensive rats. All results were analyzed statistically using two-way ANOVA followed by the Bonferrani test using GraphPad Prism. Data is presented as mean ± SEM and *** *p* < 0.001 in relation to the control group, whereas a = *p* < 0.001 in comparison with the fructose-treated group.

Adrenalin, phenylephrine, serotonin, and angiotensin II significantly increased the mean arterial pressure (MAP) in fructose hypertensive rats in comparison with the control group. Interestingly, butanolic fraction (50, 100 mg/kg) (*p* < 0.01–0.001) attenuate the increase in MAP by Adr, 5-HT, Phe, and Ang II (Figure 2).

### 2.3. DB-B-Prevented Increase in Body Weight in Fructose-Fed Rats

**Figure 3:**
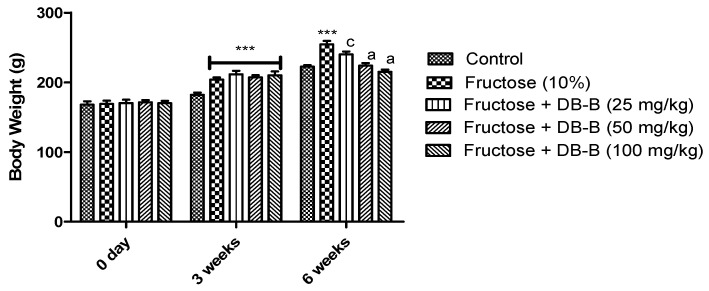
Butanolic fraction of *D. brunonianum* caused a decrease in the body weight of fructose hypertensive anesthetized rats. Data was analyzed statistically using two-way ANOVA followed by the Bonferrani test. Results are presented as mean ± SEM (n = 5) where *** = *p* < 0.001 compared with the control, while a = *p* < 0.001 and c = *p* < 0.05 compared with fructose (10%).

Chronic administration of fructose (10%) increased the body weight of the disease control group. The treatment showed a reduction in body weight as compared with the disease control group (Figure 3).

### 2.4. DB-B-Mediated Alteration in Metabolic Abnormalities Triggered by Fructose

#### 2.4.1. Reversal of Fructose-Elicited Dyslipidemia in Rats

**Figure 4:**
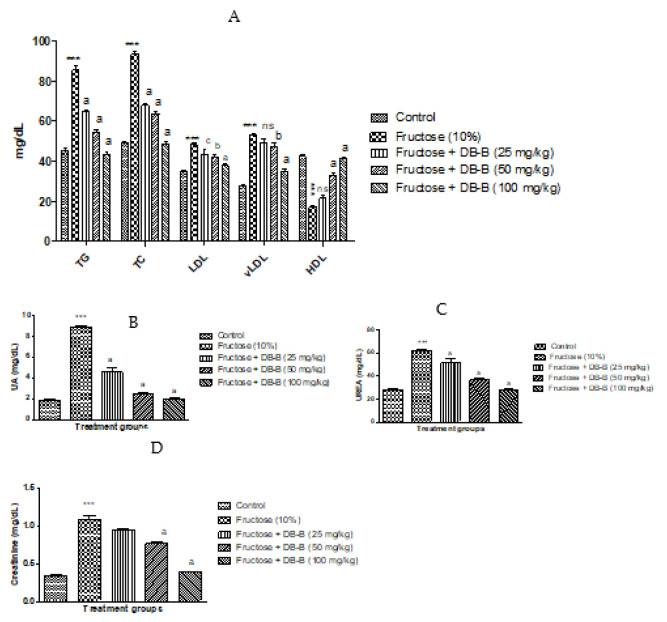
Amelioration of fructose-linked metabolic disturbances by oral administration of DB-B: (**A**) alteration in lipid profile, (**B**) decrease in level of uric acid, (**C**) level of urea, (**D**) decrease in level of creatinine. Each point is presented as mean ± SEM of five values where *** = *p* < 0.001 as compared with the control, while a = *p* < 0.001, b = *p* < 0.01, c = *p* < 0.05, and ns = non-significant in comparison with the results of the fructose (10%) group. Here, UA: uric acid, DB-B: butanolic fraction of *D. brunonianum*, TG: triglyceride, TC: total cholesterol, LDL: low-density lipoprotein, vLDL: very low-density lipoprotein, and HDL: high-density lipoprotein.

The findings of the present study revealed that there was an increase in the lipid profile of fructose-fed rats compared with the control group, while the level of HDL was significantly decreased in these rats. The increased level of triglyceride was significantly (*p* < 0.001) lowered by oral treatment with DB-B (25, 50, 100 mg/kg). The magnitude of total cholesterol was significantly (*p* < 0.01–0.001) attenuated by all doses of DB-B. Similarly, DB-B induced a profound (*p* < 0.05–0.001) decrease in LDL and vLDL. All doses of DB-B exerted a marked increase in the serum level of HDL. A total of 25 mg/kg of DB-B induced a slight modification in the level of vLDL and HDL, but that was statistically non-significant (Figure 4A).

#### 2.4.2. Decrease in Level of Uric Acid, Urea, and Creatinine in the Blood of Fructose-Fed Hypertensive Rats

Current findings presented the significant rise in the uric acid level in the blood of fructose-fed rats while DB-B (25, 50, 100 mg/kg) caused a remarkable decrease (*p* < 0.01–0.001) in the level of uric acid as shown in Figure 4B. As shown in Figure 4C, the level of urea in the blood of rats in the control was 27.906 ± 0.815 mg/dL, which was significantly increased after the chronic administration of fructose for a period of 6 weeks that was 61.796 ± 0.670 mg/dL. Treatment of DB-B caused a prominent (*p* < 0.05–0.001) decrease in the level of urea. A similar dose-dependent fall in the creatinine level was observed in fructose hypertensive rats after the oral administration of DB-B (Figure 4D).

#### 2.4.3. Reversal of Glucose Intolerance and Hyperinsulinemia

**Figure 5:**
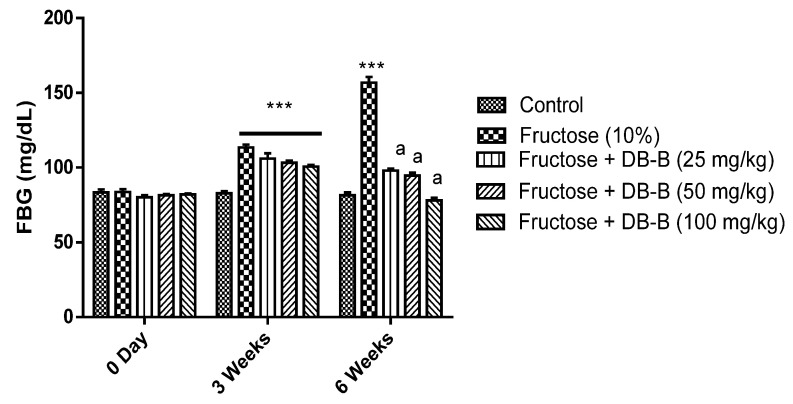
*D. brunonianum* reversed the fructose-induced rise in the level of blood glucose. Each bar is presented as mean ± SEM of five values where *** = *p* < 0.001 as compared with the control, while a = *p* < 0.001, in comparison with the results of the fructose (10%) group.

**Figure 6:**
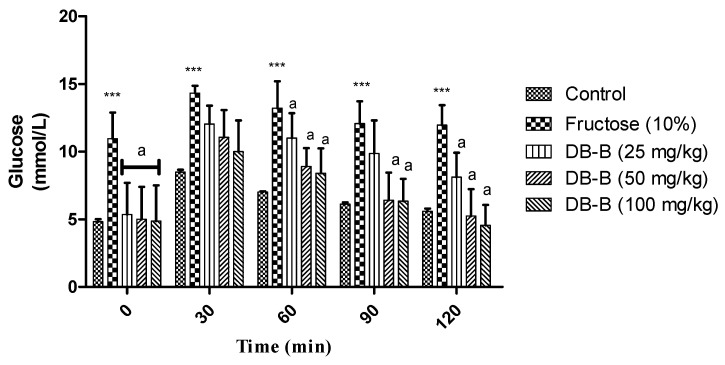
DB-B induced a decrease in glucose intolerance in fructose-fed hypertensive rats. Data was analyzed statistically using two-way ANOVA followed by the Bonferrani test. Results are expressed as mean ± SEM (n = 5) where a = *p* < 0.001 with respect to fructose (10%), whereas *** = *p* < 0.001 with respect to the control.

In fructose-fed rats, an increase in fasting blood glucose was recorded (113.4 ± 1.887 and 155 ± 5.0 mg/dl, respectively). However, the treatment with DB-B showed a significant (*p* < 0.05–0.001) reduction in the level of FBG (Figure 5). DB-B exerted the significant decline in blood-glucose intolerance (Figure 6).

**Figure 7:**
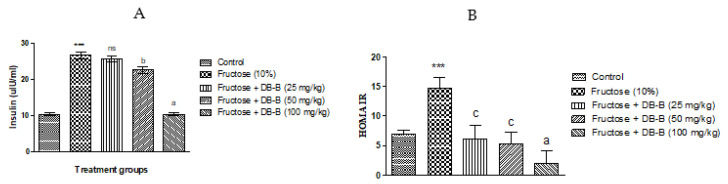
*DB-B* caused a decrease in serum insulin and HOMA-IR in fructose-fed rats: (**A**) decrease in serum insulin, and (**B**) HOMA-IR. Results were analyzed statistically using one-way ANOVA followed by the Bonferrani test. Results are plotted as mean ± SEM (n = 5) where a = *p* < 0.001 and b = *p* < 0.01, c = *p* < 0.05, and ns = non-significant when compared with fructose (10%), whereas *** = *p* < 0.001 compared with the control.

Furthermore, DB-B in doses of 50 and 100 mg/kg inverted the rise in insulin level in fructose-fed rats. (Figure 7A).

The homeostatic model of the assessment of insulin resistance offered that IR was more pronounced in the disease control group and it was significantly (*p* < 0.05–0.001) decreased in DB-B-treated rats (Figure 7B). 

#### 2.4.4. *D. brunonianum*-Evoked Protective Effect on Oxidative Stress Marker in Fructose-Treated Hypertensive Rats

**Figure 8:**
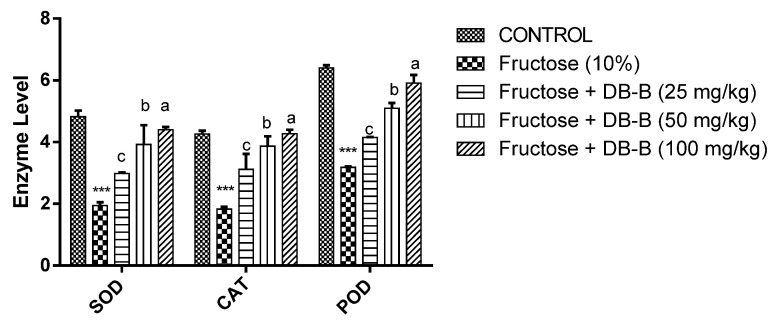
DB-B-elicited restoration of enzymes of the defense system altered by fructose All results were analyzed statistically using two-way ANOVA followed by the Bonferrani test. Data is presented as mean ± SEM of n = 5. Herein, *** represents *p* < 0.001 in comparison with the control group, whereas a = *p* < 0.001, b = *p* < 0.005, and c = *p* < 0.05 in relation to the fructose (10%) group. DB-B = butanolic fraction of *D. brunonianum*.

As presented in Figure 8, the findings of this study showed that the level of markers of oxidative stress were significantly (*p* < 0.001) increased in fructose-treated groups. The treatment significantly reinstated the levels of these enzymes. 

#### 2.4.5. *D. brunonianum*-Induced Reversal of Acetylcholine-Mediated Vasorelaxation in PE Preconstricted Aortic Tissues

**Figure 9:**
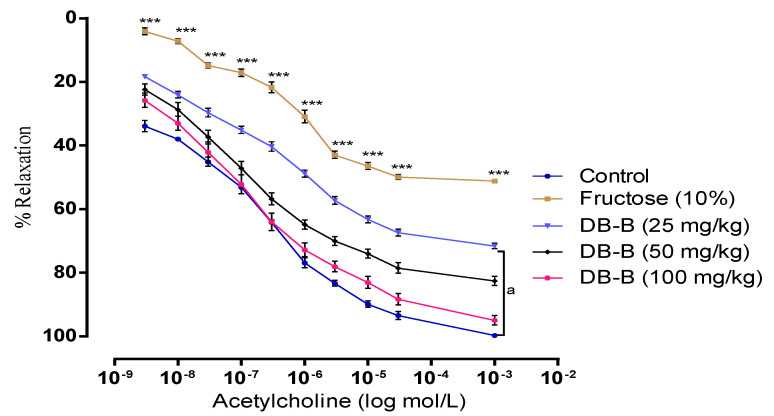
DB-B-mediated reversal of acetylcholine-evoked vasorelaxation in PE preconstricted aortic tissues. Data obtained was analyzed statistically using two-way ANOVA followed by the Bonferrani test on GraphPad Prism. Here, each point represents the mean ± SEM of n = 5. *** represents *p* < 0.001 in comparison with the control group, whereas a = *p* < 0.001 with respect to the fructose (10%) group. DB-B = butanolic fraction of *D. brunonianum*.

The findings of the current investigation had clearly revealed that oral administration of DB-B (25, 50, 100 mg/kg) and DB-Aq (100, 200, 400 mg/kg) protected endothelium from damage as in these groups acetylcholine relaxed the aortic tissue more significantly compared with the control group who only received fructose (*p* < 0.05–0.001) (Figure 9).

### 2.5. Phytochemical Analysis of DB-B Using LC-MS

**Figure 10:**
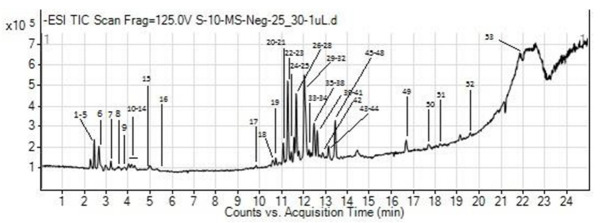
LC–MS chromatogram of DB–B.

LC-MS analysis of DB-B revealed the presence of 53 active moieties belonging to different chemical classes. The major compounds found in the chemical analysis of DB-B belonged to flavonoid, phenol, alkaloid, and tannins classes (Figure 10 and Table 1).

## 3. Discussion

A steady increase in blood pressure was recorded in this appraisal on the chronic treatment of fructose for a period of six weeks. Furthermore, findings of our study revealed that butanolic fraction of *D. brunonianum* exerted a prominent decrease in the fructose-induced elevation in blood pressure. 

Results of the current experiment showed that response to the intravenous injection of sympathomimetic agents was much reduced in rats treated with DB-B. Hence, it could be proposed that this curative effect might be associated with the sympathetic blockade by DB-B. Data obtained in these experiments has been supported by several similar studies, such as a research conducted on rats subjected to chemical sympathectomy that showed that a functional sympathetic nervous system is crucial for the development of fructose-elicited hypertension [12]. Several lines of evidence support this speculation that hypertension elicited in fructose-fed rats results from an increased degree of insulin resistance [13,14]. The findings of the present study showed that the oral administration of DB-B showed a deterioration in the effect of fructose on the level of insulin in fructose-treated rats. This probably explains that the blood-pressure-lowering effect offered by DB-B may be associated, at least in part, to preventing/reversing fructose-mediated insulin resistance. The results of the present study are similar to previously published studies [15,16,17,18]. In addition, a variety of antihypertensive agents successfully treated the high blood pressure accompanied with hyperinsulinemia [19], supporting the value of the aboriginal plant tested in this study. 

Meanwhile, we analyzed the level of lipids in the serum of DB-B-treated fructose hypertensive rats. We found that DB-B reversed and prevented fructose-linked hyperlipidemia. In addition, there are several evidences in the literature which implicate that dietary fructose directly converts to fatty acids and then to triglycerides and cholesterol, supporting the results of our study [20,21]. Coumaric acid was identified in DB-B through LC-MS analysis; in early reports, it has been demonstrated that coumaric acid decreased the level of low-density lipoprotein and, therefore, it could be proposed that the lipid lowering effect of this plant may partially be due to the presence of this compound [22].

Hyperuricemia is considered to be one of the major contributing factors in the development of fructose-induced hypertension [23]. Considering this hypothesis, we wished to evaluate the serum level of uric acid in fructose- and DB-B-treated rats. Interestingly, data obtained showed a marked elevation in the level of serum uric acid on the oral administration of fructose; however, DB-B efficiently abrogated the increase in the magnitude of uric acid. In agreement with our results, previously, it has been reported that the raised level of uric acid due to the over-feeding of fructose leads to an impaired endothelial function which will ultimately causes vasocostriction and high blood pressure [24,25]. Earlier, it was observed by different investigators that the serum level of creatinine and urea was disturbed by the chronic intake of fructose, and different natural and synthetic products successfully abolished this effect of fructose along with other metabolic perturbations [26]. Furthermore, it has been established that rutin contributed to lowering the serum level of glucose, urea, and creatinine, as chemical analysis of DB-B evidenced the presence of derivative of rutin; thus, it could be deduced that rutin may partially contribute to the pharmacological effects of DB-B [27]. We further extend our study to investigate the effect of fructose treatment on the vascular endothelium of hypertensive rats. In this way, our results suggested that the endothelial function was significantly compromised in rats feeding on fructose since the vasorelaxant effect of acetylcholine was abolished in phenylephrine-contracted aortic tissues dissected from fructose hypertensive rats; these findings are in compliance with the previous reports that there is an impairment in endothelium-mediated vasodilatation in models of metabolic syndrome [28,29,30,31]. Meanwhile, the concurrent oral administration of DB-B significantly increased the ach-evoked vasorelaxation in fructose-fed rats; therefore, we can hypothesize that the antihypertensive effect of these plants in fructose-fed rats may likely be, at least, in part associated with its vasoprotective effect. Dehydroascorbic acid has been found in DB-B, and ascorbic acid has been reported for its protective effect on endothelium. Therefore, it could be assumed that this active principle could be responsible, at least in part, for the protective effect of DB-B [32]. In addition, it has also been reported that apigenin regulates glucose, lipid metabolism, and associated endothelial dysfunction [33]. Hence, the restoration of the endothelial function with the treatment of DB-B may partially be due to the presence of apigenin.

In addition to the above-mentioned parameters, it has been clearly presented by previous research that high exposure to fructose is associated with the over-production of reactive oxygen species, suggesting that the development of metabolic disorders in humans is due to the consumption of sugar beverages and fructose-rich diets [34,35]. In the present study, the level of SOD (superoxide dismutase) was recorded as in a normal range in rats receiving both fructose and DB-B; however, the activity of serum SOD was significantly lowered in rats feeding only on fructose. It has been revealed in previous reports that SOD is involved in scavenging the O^−2^ free radical and, thus, helps in reducing blood pressure [36]. Furthermore, treatment with DB-B significantly blunted the deleterious effect of fructose on POD (peroxidase) and CAT (catalase), the supportive enzymes of the defense system involved in the catalysis of hydrogen per oxide and O_2_. Chemical analysis of DB-B revealed the presence of a large number flavonoids, and it has been well recognized that flavonoids exert antioxidant effects [37]. Robinetin 3-rutinoside, kaempferol 3-(6″-acetylglucoside)-7-glucoside, coumaric acid, malic acid, scopoline, and dehydroascorbic acid were found in DB-B. These compounds have been reported for their antioxidant potential; therefore, it could be proposed that the preventive effect of DB-B on oxidative stress may likely be due to these bioactive constituents [38,39,40,41]. Bergenin and the derivative of apigenin were identified in DB-B, and earlier studies evidenced the antioxidant potential of apigenin and bergenin; thus, it could be linked with the antioxidant activity of DB-B [42,43]. Therefore, on the basis of previous studies, it could be deduced that the phytochemicals may, at least in part, play a role in the antioxidant activity of DB-B, and it could be presented that this antioxidant activity of DB-B may be responsible for their antihypertensive effect in fructose-fed rats.

## 4. Material and Methods

### 4.1. Animals

Sprague dawley male rats weighing 170–250 g were housed in an animal house at the University of Sargodha (UOS), Sargodha, Pakistan, and maintained at a standard diet and conditions. Before the commencement of the experiments, ethical clearance for handling and experimentation on animals was obtained from the Institutional Animal Ethics Committee, University of Sargodha (Approval no. IAEC/UOS/2016/35). Additionally, all protocols complied with the declarations of the National Research Council [11].

### 4.2. Chemicals and Drugs Used

All the drugs and chemicals used for the experiments were of standard analytical grade. 

### 4.3. Plant Collection and Preparation of Extract

Aerial parts of *Delphinium brunonianum* were collected from Shinkyote Gilgit Baltistan in the months of June to July, 2016, and authenticated by Dr Sher Wali Khan, Assistant professor in the Department of Botany, Karakoram International University, Gilgit Baltistan. Specimens of the plant catalogued as DB-16-11 were preserved in the herbarium of the College of Pharmacy, University of Sargodha. In order to prepare the crude extract, powdered aerial parts of *Delphinium brunonianum* were macerated in an aqueous ethanol mixture (30:70) and extracted thrice (72 h each). The obtained extract was filtered, and the solvent was eliminated by a rotary evaporator. The obtained extract was stored in a tightly closed container. Afterwards, the aqueous ethanolic extract (crude extract) was subjected to subsequent fractionation with organic solvents [10]. Butanolic fraction (DB-B; yield 15%), used in this study, was stored in capped containers and maintained at 4 °C, until use for analysis. The dose of DB-B was freshly prepared in distilled water at the time of administration. All doses (according to body weight) were administered via oral route.

### 4.4. Effect of Butanolic Fraction of Delphinium brunonianum on Fructose-Induced Hypertension and Metabolic Alterations in Rats

Rats in all groups, except the control group, were given 10% fructose in drinking water for the first 3 weeks, whereas the treatment groups received the DB-B (25, 50, 100 mg/kg) for the next 3 weeks (in total 6 weeks of study); however, the rats in the fructose-treated group were fed on only fructose. At the end of 6 weeks, blood pressure was measured using an invasive blood pressure measuring technique. In order to evaluate the effect on sympathetic over-activity, rats from all groups were evaluated for vascular reactivity to various agents such as adrenaline (1 g/kg), phenylephrine (1 g/kg), and angiotensin II (25 ng/kg), and serotonin (1 g/kg) was also computed to scrutinize the function of the adrenergic nervous system [44,45,46].

### 4.5. Alteration in Fructose-Triggered Metabolic Abnormalities

The fresh serum from rats of all groups was subjected to assess level of lipids, insulin, uric acid, urea, creatinine using standard biochemical kits. Furthermore, body weight and fasting blood glucose was measured at different intervals during the whole study [11].

### 4.6. Evaluation of DB-B-Mediated Reversal of Glucose Intolerance and Insulin Resistance in Fructose-Fed Rats

At the end of the foregoing experiment, rats were deprived of food for 12 h and the oral glucose tolerance test was conducted using glucometer. After the glucose load (50% solution of glucose (1 mL/100 g B.W)), the level of blood glucose was measured at 0, 30, 60, 90, and 120 min. Later on, HOMA-IR (homeostatic model assessment-insulin resistance) was calculated by the equation given in [47]:

insulin (u/mL) × glucose (mM/L)/22.5

### 4.7. Analysis of Alteration in the Level of Enzymes of the Oxidative System

For this purpose, serum level of catalase, superoxide dismutase, and peroxidase were estimated in blood samples of all rats from all treatment and control groups by adopting the method described by Younis and coworkers [48].

### 4.8. Effect of Butanolic Fraction of D. brunonianum on Fructos- Mediated Dysfunction of Vascular Endothelium

To delineate the effect of DB-B on fructose-mediated endothelium damage, the thoracic aorta of all animals was observed for vascular reactivity. Vascular reactivity of aortic rings with intact endothelium was assessed by relaxation induced by acetylcholine against the pre-contraction of phenylephrine (1 µM) [18,49].

### 4.9. Phytochemical Analysis of DB-B

Phytochemicals present in DB-B were analyzed through RP UHPLC-MS following the method of Saleem et al. [50]. Briefly, UHPLC of an Agilent 1290 Infinity liquid chromatography system accompanied with Agilent 6520 Accurate-Mass Q-TOF mass spectrometer with dual ESI source was used in this study, whereas the column used was Agilent Zorbax Eclipse XDB-C18, narrow-bore 2.1 × 150 mm (3.5 μm P/N: 930990-902), which was kept at 25 °C. The auto-sampler was maintained at 4 °C. A total of 1.0 μL of the sample was injected at a flow rate of 0.5 mL/min, while 0.1% formic acid in water (A) and 0.1% formic acid in acetonitrile (B) were used as the mobile phase. The sample was run for 25 min and an extra 5 min were given. Full-scan mass spectrometry analysis was conducted on *m*/*z* 100–1000, applying a negative electrospray ion source. The recorded results were processed with Agilent Mass Hunter Qualitative Analysis B.05.00 (Metabolomics-2017-00004.m). The compounds were identified by Search Database: METLIN_AM_PCDL-Ne 170502.cdb, with parameters such as match tolerance: 5 ppm, positive ions: +H, +Na, +NH_4_, and negative ions: H.

### 4.10. Statistical Analysis

Data was analyzed using one-way/two-way (ANOVA) followed by an appropriate post-test, either Dunnet or Bonfferoni, using GraphPad Prism. *p* < 0.05 were considered as statistically significant.

## 5. Conclusions

In conclusion, the findings of the current study proposed that butanolic fraction of *Delphinium brunoninaum* was found to restore the fructose-mediated hypertension and metabolic abnormalities. DB-B significantly prevented sympathetic over-activity and insulin resistance. As described in the current study, DB-B could efficiently restore the oxidative enzymes and endothelium integrity. Hence, butanolic fraction of *Delphinium brunonianum* has the potential to prevent the fructose-mediated metabolic abnormalities, and it could serve as a promising source for the development of new therapeutically useful compounds. Nonetheless, further investigation and the isolation of active principles identified in LC-MS analysis is required to validate the mechanism of action

## Figures and Tables

**Table 1:** List of compounds identified by LC-MS analysis of DB-B.

S.NO	RT (min)	Base Peak *m*/*z*	Peak Height	Proposed Compound	Molecular Formula	Molecular Mass	Volume
1	2.652	217.0481	46,409	bis(4-fluorophenyl)-Methanone	C_13_ H_8_ F_2_ O	218.0534	187,240
2	2.668	215.0321	5966	2-C-Methyl-D-erythritol 4-phosphate	C_5_ H_13_ O_7_ P	216.0395	24,028
3	2.672	181.0712	18,085	D-Sorbitol	C_6_ H_14_ O_6_	182.0785	76,215
4	2.697	179.0557	9855	L-Galactose	C_6_ H_12_ O_6_	180.0631	48,970
5	2.753	135.0299	8636	D-threonic acid	C_4_ H_8_ O_5_	136.0371	40,365
6	2.972	366.1153	12,110	Met Ser Met	C_13_ H_25_ N_3_ O_5_ S_2_	367.1229	76,009
7	3.214	133.0147	48,234	D-(+)-Malic acid	C_4_ H_6_ O_5_	134.022	233,663
8	3.57	290.0883	7253	Sarmentosin epoxide	C_11_ H_17_ N O_8_	291.0956	75,515
9	3.832	253.0929	7434	Galactosylglycerol	C_9_ H_18_ O_8_	254.1001	46,868
10	4.028	128.0356	34,200	N-Acryloylglycine	C_5_ H_7_ N O_3_	129.0429	208,646
11	4.152	243.0619	7285	Uridine	C_9_ H_12_ N_2_ O_6_	244.0691	49,247
12	4.292	188.0562	8154	Glutarylglycine	C_7_ H_11_ N O_5_	189.0635	51,869
13	4.292	129.0198	6037	Glutaconic acid	C_5_ H_6_ O_4_	130.027	39,902
14	4.293	173.0091	14,206	Dehydroascorbic acid	C_6_ H_6_ O_6_	174.0164	91,494
15	4.995	117.0195	20,991	Erythrono-1,4-lactone	C_4_ H_6_ O_4_	118.0268	139,568
16	5.681	129.0194	6310	Glutaconic acid	C_5_ H_6_ O_4_	130.0267	50,742
17	9.83	131.0355	11,447	3-Hydroxy-3-methyl-2-oxo-Butyric acid	C_5_ H_8_ O_4_	132.0428	58,944
18	10.632	293.1244	10,448	Ethyl (S)-3-hydroxybutyrate glucoside	C_12_ H_22_ O_8_	294.1317	43,798
19	10.732	327.0723	17,123	Bergenin	C_14_ H_16_ O_9_	328.0795	84,427
20	11.07	353.0885	54,518	Scopolin	C_16_ H_18_ O_9_	354.0957	369,833
21	11.071	467.0816	6864	Castamollissin	C_20_ H_20_ O_13_	468.0887	34,751
22	11.277	609.1477	130,491	Robinetin 3-rutinoside	C_27_ H_30_ O_16_	610.1547	1,136,964
23	11.283	175.0611	6144	3-propylmalic acid	C_7_ H_12_ O_5_	176.0685	28,288
24	11.437	340.1558	11,223	Carboxyterbinafine derivative2	C_20_ H_23_ N O_4_	341.163	68,196
25	11.437	403.151	10,458	Desmethylnimodipine	C_20_ H_24_ N_2_ O_7_	404.1586	55,189
26	11.565	593.1513	47,204	Luteolin 7-rhamnosyl(1- > 6)galactoside	C_27_ H_30_ O_15_	594.1586	266,559
27	11.662	651.157	105,640	Kaempferol 3-(6″-acetylglucoside)-7-glucoside	C_29_ H_32_ O_17_	652.1642	849,836
28	11.78	609.1452	15,662	Robinetin 3-rutinoside	C_27_ H_30_ O_16_	610.1524	90,280
29	12.016	295.0453	38,925	Mono-trans-p-coumaroylmesotartaric acid	C_13_ H_12_ O_8_	296.0527	394,426
30	12.051	635.1622	25,444	Fujikinetin 7-O-laminaribioside	C_29_ H_32_ O_16_	636.1699	152,900
31	12.052	651.1574	86,304	Kaempferol 3-(6″-acetylglucoside)-7-glucoside	C_29_ H_32_ O_17_	652.1647	852,487
32	12.089	186.1143	12,154	KAPA	C_9_ H_17_ N O_3_	187.1216	70,741
33	12.231	593.1528	5324	Luteolin 7-rhamnosyl(1- > 6)galactoside	C_27_ H_30_ O_15_	594.16	28,075
34	12.254	463.0881	19,178	8-Hydroxyluteolin 8-glucoside	C_21_ H_20_ O_12_	464.0951	108,984
35	12.336	635.1623	13,934	Fujikinetin 7-O-laminaribioside	C_29_ H_32_ O_16_	636.1698	78,444
36	12.448	635.1623	9397	Fujikinetin 7-O-laminaribioside	C_29_ H_32_ O_16_	636.1692	52,438
37	12.473	693.1673	49,263	Isoscutellarein 7-(6‴-acetylallosyl-(1- > 2)-6″-acetylglucoside)	C_31_ H_34_ O_18_	694.1745	405,976
38	12.478	555.1977	5318	punaglandin 1	C_27_ H_37_ Cl O_10_	556.2062	33,134
39	12.62	693.1672	41,258	Isoscutellarein 7-(6″-acetylallosyl-(1- > 2)-6″-acetylglucoside)	C_31_ H_34_ O_18_	694.1743	287,244
40	12.717	597.2099	8960	12S-acetoxy-punaglandin 1	C_29_ H_39_ Cl O_11_	598.2179	47,677
41	12.766	163.0399	7568	m-Coumaric acid	C_9_ H_8_ O_3_	164.047	33,265
42	12.859	677.171	11,272	Apigenin 7-(4″,6″-diacetylalloside)-4′-alloside	C_31_ H_34_ O_17_	678.1785	66,085
43	13.133	677.1739	7170	Apigenin 7-(4″,6″-diacetylalloside)-4′-alloside	C_31_ H_34_ O_17_	678.1804	40,991
44	13.137	447.0937	11,848	6-Hydroxyluteolin 5-rhamnoside	C_21_ H_20_ O_11_	448.1007	70,221
45	13.431	121.029	6423	3-Hydroxybenzaldehyde	C_7_ H_6_ O_2_	122.0362	28,930
46	13.437	237.0393	6940	Dipyrocetyl	C_11_ H_10_ O_6_	238.0465	31,193
47	13.438	699.3497	96,124	Septentriodine	C_37_ H_52_ N_2_ O_11_	700.3569	634,267
48	13.705	269.1032	7341	Idebenone Metabolite (Benzenebutanoic acid,2,5-dihydroxy-3,4-dimethoxy-6-methyl-)	C_13_ H_18_ O_6_	270.1104	40,569
49	16.669	221.1184	34,676	(6S)-dehydrovomifoliol	C_13_ H_18_ O_3_	222.1257	211,059
50	17.71	293.1769	12,618	Gingerol	C_17_ H_26_ O_4_	294.1842	81,405
51	18.219	309.1711	10,585	methyl 8-[2-(2-formyl-vinyl)-3-hydroxy-5-oxo-cyclopentyl]-octanoate	C_17_ H_26_ O_5_	310.1785	70,372
52	19.584	277.1809	7965	6-Paradol	C_17_ H_26_ O_3_	278.1881	50,566
53	21.808	265.1489	22,964	Lauryl hydrogen sulfate	C_12_ H_26_ O_4_ S	266.1563	362,120

## Data Availability

The data presented in this study are available in article.
